# Biochemical Predictors and Clinical Characteristics for the Development of Cytopenia and Bone Marrow Involvement Among Patients With Primary Hyperparathyroidism

**DOI:** 10.1155/ije/4158433

**Published:** 2026-07-27

**Authors:** Omer Abdelfadiel, Mohamed Abdalla, Ian Louis Ross

**Affiliations:** ^1^ Department of Endocrinology, Burjeel Royal Hospital and Burjeel Day Surgery Centre, Al Ain, Abu Dhabi, United Arab Emirates; ^2^ Dasman Diabetes Institute, Kuwait City, Kuwait, dasmaninstitute.org; ^3^ Division of Endocrinology, Department of Medicine, Groote Schuur Hospital, University of Cape Town, Cape Town, South Africa, uct.ac.za

**Keywords:** bone marrow fibrosis, cytopenia, myelofibrosis, parathyroid hormone, primary hyperparathyroidism

## Abstract

**Background:**

The clinical features associated with primary hyperparathyroidism (PHPT) giving rise to bone marrow fibrosis are poorly documented. We hypothesised that the severity of PHPT may contribute to the development of this complication.

**Methods:**

We identified two clinical cases managed within our clinical service and reviewed case reports and cohort studies using search terms, *inter alia,* ‘primary hyperparathyroidism,’ ‘pancytopenia,’ ‘myelofibrosis’ and ‘bone marrow fibrosis,’ utilising PubMed, Google Scholar, Microsoft Academic Search and Web of Science. We excluded patients with chronic renal failure and/or secondary hyperparathyroidism. Studies lacking a histological assessment of bone marrow were also excluded.

**Results:**

We identified a total of 41 reports that satisfied our search criteria, comprising individual cases and three previously reported cohorts. Of these, 27 cases were excluded as they had secondary hyperparathyroidism from renal failure, or because an alternative aetiology for bone marrow fibrosis, such as toxin exposure, was identified. In addition, one large cohort of patients with PHPT and cytopenia was excluded due to the absence of a bone marrow biopsy and missing key biochemical and skeletal data. The included reports consisted of 11 individual cases of bone marrow fibrosis associated with PHPT, along with 2 small cohorts of 17 and 8 patients, respectively. Common clinical features across the 11 case reports included markedly elevated parathyroid hormone (PTH) levels (mean ± SD: 130 ± 95.8 pmol/L; normal range: 1.1–6.9 pmol/L), low serum 25‐hydroxyvitamin D (median [IQR]: 20.0 [17.0–30.0] nmol/L; normal range: 75–150 nmol/L) and significantly elevated alkaline phosphatase (ALP) (median [IQR]: 361 [293–1372]) U/L; normal range: 30–120 U/L). Bone marrow fibrosis in this instance is reversible following excision of the culprit parathyroid lesion.

**Conclusion:**

Bone marrow fibrosis associated with PHPT occurs predominantly in the setting of severe biochemical disease, characterised by markedly elevated PTH and ALP levels and may present with variable patterns of cytopenia. While symptom duration varies widely, disease severity rather than chronicity appears to be the principal determinant for bone marrow involvement. Haematological abnormalities are reversible following parathyroidectomy, highlighting the importance of timely diagnosis and intervention.

## 1. Introduction

Primary hyperparathyroidism (PHPT) is a common endocrine disorder that has evolved from being primarily symptomatic, associated with hypercalcaemia and fractures, to a condition that is largely asymptomatic. The incidence of PHPT has increased over the last several decades, attributable to enhanced calcium screening and assessment of bone mineral density (BMD). PHPT may present at any age, but the majority of cases occur in patients over the age of 50 years, with more women affected than men [1]. The most common pathological finding in PHPT is a solitary parathyroid adenoma occurring in approximately 85%–90% of cases. Multiglandular disease, including hyperplasia and multiple adenomas, accounts for 10%–15%, whereas parathyroid carcinoma is rare, occurring in around 1% [2]. The severity of hypercalcaemia associated with PHPT may vary from being normal or mildly elevated and asymptomatic, to a parathyroid crisis, in which the serum calcium is very high and life‐threatening [3].

The typical manifestations of PHPT include skeletal involvement, nephrolithiasis and neuropsychiatric symptoms such as depression and anxiety [3]. Skeletal disease ranges from reduced BMD and fragility fractures to osteitis fibrosa cystica (OFC), the classical and pathognomonic radiographic manifestation of advanced PHPT, characterised by subperiosteal bone resorption, bone cysts and brown tumours [4]. In addition, PHPT has been associated with increased cardiovascular risk, including hypertension, endothelial dysfunction, arrhythmias and left ventricular hypertrophy [5].

By contrast, cytopenia and myelofibrosis are very unusual manifestations of PHPT and have been reported in only a limited number of case reports and small cohort studies [6–17]. Secondary hyperparathyroidism (HPT) from chronic renal failure, on the other hand, may induce bone marrow pathology far more commonly, often associated with renal osteodystrophy [18, 19]. The association between myelofibrosis and secondary HPT due to end‐stage renal disease (ESRD) has been well recognised since the 1970s [20]. Myelofibrosis has been reported in patients with secondary HPT due to vitamin D insufficiency. Cases of secondary HPT and myelofibrosis resulting from severe vitamin D deficiency after gastric bypass surgery for severe obesity have been described [21]. This association has also been reported in the context of osteomalacia and coeliac disease [22].

The entity of PHPT‐associated bone marrow fibrosis (PHPT‐ABMF) remains poorly characterised due to its rarity. Although a large cohort by Kır and Polat reported cytopenias in patients with PHPT, the absence of bone marrow histology, together with the lack of information on vitamin D, alkaline phosphatase (ALP) and skeletal imaging, limited direct evaluation of this association [23]. Moreover, the risk factors associated with PHPT‐ABMF are poorly documented. We therefore sought to identify clinical associations and predictive factors, hypothesising that the severity of PHPT may play a contributory role.

## 2. Methods

### 2.1. Research and Ethics

The two cases under our care provided written informed consent, whereas the remaining cases were extracted from the previously published case reports and small series. The University of Cape Town, Faculty of Health Sciences Research and Ethics Committee waived the need for a full research ethics application but required signed informed consent.

All cases of PHPT with myelofibrosis and/or cytopenia were identified using the following search terms: “primary hyperparathyroidism,’ ‘pancytopenia,’ ‘myelofibrosis,’ ‘bone marrow fibrosis’ and ‘parathyroid adenoma,’ which were combined in all permutations and entered into the following search engines: PubMed, Google Scholar, Microsoft Academic Search and Web of Science.

Cytopenia was defined as a reduction in one or more peripheral blood lineages, including anaemia, leucopenia and thrombocytopenia, according to standard laboratory reference ranges. Cytopenia cases were excluded if they had a history of renal disease, a diagnosis of renal osteodystrophy or metastatic disease to bone. Cases were also excluded if there was an identifiable cause for cytopenia other than PHPT, including medication or exposure to hydrocarbons known to be toxic to bone marrow. We also excluded studies that did not report bone marrow histology. All biochemical results were converted to SI units to ensure consistency and comparability.

### 2.2. Statistics

The statistical analyses employed in this case series were primarily descriptive, aimed at summarising the clinical and laboratory characteristics of the 11 cases with PHPT‐associated cytopenia and comparing them with two previously published cohorts: Bhadada (*n* = 8) and Boxer (*n* = 17). Statistical analyses were conducted using SPSS Version 30 (IBM Corp., Armonk, NY, USA, 2024).

Continuous variables were assessed for normality using the Shapiro–Wilk test and for homogeneity of variances using Levene’s test. Normally distributed variables (haemoglobin, calcium, parathyroid hormone [PTH] and phosphate) were summarised as mean ± standard deviation (SD). Non‐normally distributed variables across all study groups (ALP and vitamin D) and in at least one study group (age and serum creatinine) were summarised as median (interquartile range [IQR]).

Global comparisons across the three groups were performed using one‐way ANOVA for normally distributed variables with homogeneous variances (serum phosphate), Welch’s ANOVA when variances were unequal (haemoglobin and serum calcium) and the Kruskal–Wallis test for variables that were non‐normally distributed in any group (age and serum creatinine).

All statistical tests were conducted as two‐tailed. Global comparisons were considered statistically significant at a *p* < 0.05. Post hoc pairwise comparisons were performed only when the corresponding global test was statistically significant. Games–Howell tests were used following Welch’s ANOVA, Bonferroni‐adjusted pairwise comparisons after one‐way ANOVA and Mann–Whitney *U* tests after Kruskal–Wallis analyses; *p*‐values from the Mann–Whitney U comparisons were adjusted using the Holm method to control for multiplicity and reduce the risk of Type I error. Detailed pairwise results are provided in Supporting Table S1.

Statistical comparison of PTH and vitamin D was restricted to the case‐report and Bhadada cohorts because the Boxer cohort reported PTH in nonstandard assay‐specific units and did not report vitamin D values; comparisons were therefore performed using the Mann–Whitney *U* test. Comparison of ALP across cohorts was not performed due to heterogeneity in units and reference ranges.

Categorical variables (sex, radiographic findings, bone marrow biopsy, parathyroid histology and haematological recovery) were presented as frequencies and percentages. Group differences were assessed using the Fisher–Freeman–Halton exact test, given small expected cell counts (< 5) and contingency tables larger than 2 × 2.

Missing data, including vitamin D levels and unavailable radiographic or histopathological reports, were explicitly acknowledged to highlight study limitations. Key findings were summarised in tables to enhance clarity and facilitate interpretation.

## 3. Results

**FIGURE 1:**
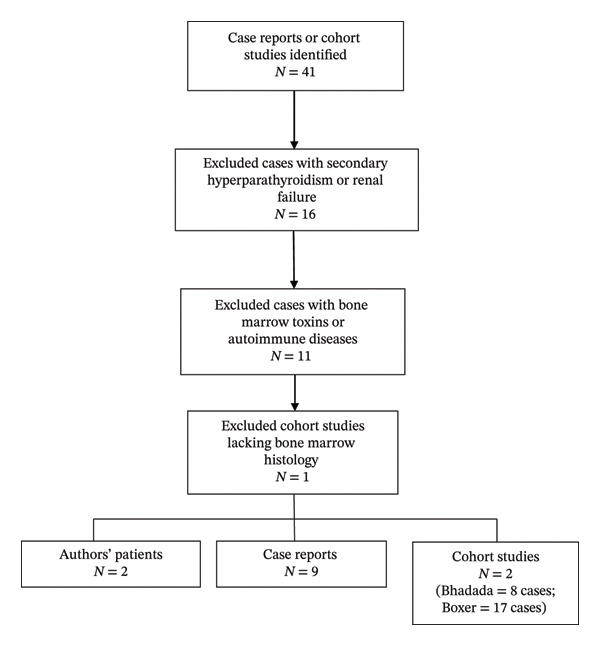
Flow diagram illustrating the identification of case reports and cohort studies describing cytopenia and bone marrow involvement in association with primary hyperparathyroidism (PHPT).

The process of identifying cases of cytopenia in association with PHPT yielded 11 individual case reports, along with two small previously published cohorts (Figure 1).

Two of the 11 case reports were contributed by our team. The first patient presented after a prolonged symptomatic course of approximately 16 months, with pancytopenia, while the second presented with isolated anaemia identified during evaluation of a 5‐week clinical course. Both patients had marked hypercalcaemia, substantially elevated PTH levels, radiological features consistent with high‐turnover skeletal disease and bone marrow fibrosis. Haematological recovery, defined as normalisation of peripheral blood counts, occurred within 2–3 months following parathyroidectomy. Notably, the postoperative course in both patients was complicated by hungry bone syndrome, reflecting the severity of their underlying skeletal involvement.

Among the 11 case reports, a predominance of the female sex was observed (7/11; 63.6%), with a median age of 41 years (IQR: 19–49). Fatigue and generalised weakness were the most commonly described symptoms. Musculoskeletal and gastrointestinal complaints were variable, while polydipsia, polyuria and flank pain were less frequent. The duration of symptoms prior to diagnosis was reported in 6 of the 11 cases and ranged from approximately 2 weeks to 36 months, with other documented durations of 5 weeks, 3 months, 6 months and 16 months. Physical examination failed to reveal clinical features common to all patients. The clinical characteristics, albeit incomplete, are shown in Table 1.

**TABLE 1:** Clinical characteristics and relevant investigations for the individual cases of PTH‐associated cytopenias.

	Case 1 (Ross et al.) [**6**]	Case 2 (Lim et al.) [**7**]	Case 3 (Akyay et al.) [**8**]	Case 4 (Gokosmanoglu et al.) [**9**]	Case 5 (Kumbasar et al.) [**10**]	**Case 6 (current case)**

Sex	Female	Female	Male	Female	Female	Male
Age at presentation	42 years	41 years	15 years	22 years	18 years	59 years
Symptoms	Severe progressive generalised weakness	Generalised weakness; arthralgia, especially knee joint pain	Generalised weakness; intermittent vomiting	Neck swelling	Bilateral knee pain; gait disturbance; fatigue	Abdominal and right flank pain; fatigue
Duration of symptoms	16 months		1 month		6 months	5 weeks
Comorbidity	Intracranial meningiomas; bipolar affective disorder		Hearing impairment (conductive hearing loss)			
Examination	Pallor; proximal muscle weakness	Mild splenomegaly	Pallor; hepatosplenomegaly	Neck lesion	Thyroid nodule; apical systolic murmur; splenomegaly, tetraparesis	Pallor; right renal angle tenderness
Full blood count: WBC (× 10^9^/L), Hb (g/dL), PLT (× 10^9^/L)	WBC 2.59 (4–10), Hb 9.2 (12–15), PLT 161 (180–400)	WBC 3.61, Hb 9.7, Hct 29%, PLT 71	WBC 2.4, Hb 6.2, Hct 21.6%, PLT 65	WBC 2.2. Hb 4.7, PLT 69	WBC 3.4, Hb 7.0, Hct 21.8% (37%–47%), PLT 155	WBC 8.14, Hb 5.3, Hct 19.4%, PLT 480
Serum creatinine (μmol/L)	88.0 (49.0–90.0)	Normal	Normal		Normal	142.0 (49.0–90.0)
Serum calcium (mmol/L)	2.48 (2.05–2.56)	2.6	3.1 (2.12–2.57)	3.0 (2.15–2.5)	3.12 (2.10–2.55)	3.73
Ionised calcium (mmol/L)		1.36 (1.13–1.32)			1.69 (1.12–1.32)	
PTH (pmol/L)	186 (1.6–6.9)	104.9 (1.4–5.7)	14.6 (1.2–5.8)	34.7 (1.6–6.9)	PTH‐C terminal 530.0 (42.4–148.5)	279.3 (1.6–6.9)
Serum phosphate (mmol/L)	0.51 (0.80–1.40)	0.7 (0.8–1.5)	0.8 (0.81–1.45)	0.71 (0.74–1.52)	0.58 (0.87–1.45)	0.82
Serum Vit D (nmol/L)	9.3 (50–250)			17.0 (74.9–199.7)		20.0 (50–150)
Serum ALP (U/L)	378 (40–120)	6.82 (0.3–0.9)^∗^	289	135 (32–104)	1937 (53–128)	361 (53–128)
Skeletal X‐ray	Osteopenia; subperiosteal erosions	Salt‐and‐pepper skull; generalised osteopenia (osteitis fibrosa cystica)		Mandible lytic lesion (brown tumour)	Resorption; trabeculation	Multiple lytic lesions
DEXA measurement	Osteoporosis	T‐Score: L1–L4 –5.10 ± 0.43, femoral neck −4.7 ± 0.68		T‐score: L1–L4 −3.4, femoral neck −3.1		
Bone marrow biopsy	Extensive osteosclerosis; dense marrow fibrosis**;** markedly reduced haemopoiesis**;** extensive bone marrow collagen fibrosis	Marked fibrosis**;** increased fibroblast activity; diffuse reticulin network**;** focal collagenisation	Hypocellular aspirate with decreased erythroid, myeloid and megakaryocyte lineages; trephine biopsy: Grades 3–4 reticulin fibrosis, increased collagen fibrosis	Pancytopenia; increased reticulin fibrosis	Hypocellular aspirate and biopsy; increased osteoblastic and fibroblastic activity; elevated reticulin fibres	Extensive intertrabecular and paratrabecular marrow fibrosis
Parathyroid histology	Adenoma	Adenoma (1.5 cm)	Adenoma	Adenoma and cyst	Adenoma (3.5 cm)	Adenoma
Complications	Severe hungry bone syndrome		Acute respiratory distress		Left kidney stones	Severe hungry bone syndrome
Haematological recovery	3 months	6 months	4 months	3 months	3 months	2 months

	Case 7 (Bhadada et al.) [**11**]	Case 8 (Huang et al.) [**12**]	Case 9 (Rajan et al.) [**13**]	Case 10 (Razavi and Alaghehbandan) [**14**]	Case 11 (Gupta et al.) [**15**]	

Sex	Female	Female	Female	Male	Male	
Age at presentation	30 years	58 years	48 years	49 years	19 years	
Symptoms	Easy fatigability	Fatigue; general malaise; polydipsia and sacrum pain	Easy fatigability and generalised weakness; lower backache and ankle pain	Polydipsia; polyuria; constipation; abdominal and lower back pain	Fragility fracture of the neck of the right femur; Right hip pain and inability to walk	
Duration of symptoms	3 months	2 weeks	36 months			
Comorbidity		Hypertension		Epilepsy (resolved postleft frontal lobe resection for childhood trauma‐related scar tissue)		
Examination	Pallor	2 cm lesion in the right lower neck		2 cm nodule on the left thyroid lobe		
Full blood count: WBC (× 10^9^/L), Hb (g/dL), PLT (× 10^9^/L), Hct (%)	Normal WBC, Hb 9.1 (12.0–15.0), PLT 55 (150–400)	WBC 6.4, Hb 7.4 (12–16), Hct 30.1%, PLT 164 (150–400)	WBC 3.0 × (4–12), Hb 6.3 (12–15), PLT 60 (150–450)	Hb 9.9	WBC 12.0, Hb 9.0, PLT 260	
Serum creatinine (μmol/L)	Normal	141.4 (44.2–132.6)	76.0 (35.4–123.8)	161.0		
Serum calcium (mmol/L)	3.37 (2.12–2.55)	4.04 (2.25–2.74)	3.87 (2.07–2.59)	3.66	4.40 (2.15–2.55)	
Ionised calcium (mmol/L)				1.9		
PTH (pmol/L)	36.8 (0.5–6.4)	111.3 (1.3–7.6 L)	220.8 (0.8–7.6)	67.6	244.0 (0.0–7.4)	
Serum phosphate (mmol/L)	0.77 (0.81–1.45)	0.84 (0.81–1.45)	0.71 (0.81–1.48)	0.60		
Serum Vit D (nmol/L)	62.4 (27.7–107.1)		20 (75–150)		30.0 (74.9–149.8)	
Serum ALP (U/L)	321 (42–128)	297 (30–120)	4132 (40–125)	bs ALP 170	806 (40–129)	
Skeletal X‐ray	Normal			Lytic lesions in the L4 vertebral body	Multiple pelvic lytic lesions	
DEXA measurement			Z‐score: lumbar spine, −2.3, total hip −2.4 and distal one‐third of the radius −4.1			
Bone marrow biopsy	Megakaryocytic thrombocytopenia; fibrosis	Moderate erythroid hypoplasia with patchy moderate reticulin fibrosis	Fibrosis	Bony trabecular remodelling and marked paratrabecular fibrosis	Peritrabecular, intertrabecular and diffuse Grade 3 fibrosis	
Available genetics						
Parathyroid histology		Adenoma	Adenoma	Adenocarcinoma	Adenoma	
Complications	Recurrent renal stones; bilateral Grade 1 hydronephrosis			Acute kidney injury	Bilateral nephrocalcinosis; fracture of the neck of the femur; pathological fracture of the right femur shaft	
Haematological recovery	2 months	12 months	3 months			

*Note:* Blank cells indicate data unavailable from the case reports. All values are converted to SI units for consistency.

Abbreviations: ALP = alkaline phosphatase; bs ALP = bone‐specific alkaline phosphatase; Hb = haemoglobin; Hct = haematocrit; PLT = platelet count; PTH = parathyroid hormone; Vit D = 25‐hydroxy vitamin D; WBC = white blood cell count.

^∗^The reported ALP level (113.7 nkat/L [5.8–15.2 nkat/L]) converts to a value well below the standard reference range (40–120 U/L), suggesting a possible error in decimal placement or in the unit used.

The mean serum calcium was 3.44 ± 0.60 mmol/L (normal range: 2.1–2.6 mmol/L), and the mean serum phosphate was 0.70 ± 0.10 mmol/L (normal range: 0.79–1.45 mmol/L). The degree of PTH elevation varied between 14.6 and 279.3 pmol/L (normal range: 1.1–6.9 pmol/L). Although serum vitamin D concentration was not recorded in most cases, available data showed frequently low levels, with a median of 20.0 nmol/L (IQR: 17.0–30.0; normal: 75–150). Serum ALP was markedly elevated in most cases, often exceeding the upper reference limit by several‐fold, with values ranging from 135 to 1937 U/L (median: 361, IQR: 293–1372; normal: 30–120 U/L).

Of the 8 cases with available skeletal radiographs, 7 showed abnormalities, including osteopenia, lytic lesions and subperiosteal resorption. Bone marrow biopsies consistently demonstrated varying degrees of fibrosis in all 11 cases. Parathyroid adenoma was the predominant histological finding, with only one case identified as parathyroid carcinoma, and no cases consistent with hyperplasia. Haematological recovery following parathyroidectomy was documented in 9 of the 11 cases, with a median recovery time of 3 months (IQR: 2.5–5.0); follow‐up data were unavailable for the remaining two cases.

We compared a summation of the 11 case reports with the two previously published cohorts reported by Bhadada et al. (*n* = 8) and Boxer et al. (*n* = 17) to determine whether our identified case reports differed from these cohorts (Table 2). A female predominance was observed among all groups (63.6%, 75.0% and 70.6%, respectively), with no significant difference between them (*p* = 0.889). The mean age at presentation differed significantly between groups (*p* = 0.002), with patients in the Boxer cohort being older than those in the other two groups.

**TABLE 2:** Comparison of clinical and biochemical data between 11 case reports and previously published cohorts.

Variable	Case reports *N* = 11	Bhadada et al. *N* = 8	Boxer et al. *N* = 17	*p* value	Test
Female sex, *n*/*N* (%)	7/11 (63.6)	6/8 (75.0)	12/17 (70.6)	0.889	Fisher–Halton
Age at presentation (years)	41 (19–49)	30.5 (25–40.5)	58.0 (48.5–65.5)	0.002	Kruskal–Wallis
Haemoglobin (g/dL)	7.6 ± 1.8	11.5 ± 0.7	9.8 ± 1.2	< 0.001	Welch’s ANOVA
Serum calcium (mmol/L)	3.44 ± 0.60	2.61 ± 0.15	3.5 ± 0.59	< 0.001	Welch’s ANOVA
PTH	130 ± 95.8 pmol/L	110.6 ± 102.6 pmol/L	99.5 ± 26.4 units	0.633^∗^	Mann–Whitney U
Serum phosphate (mmol/L)	0.70 ± 0.10	0.96 ± 0.11	0.84 ± 0.19	0.013	One‐way ANOVA
Serum creatinine (μmol/L)	141.4 (82–151.5)	74.9 (50–74.98)	123.8 (92.8–163.6)	0.002	Kruskal–Wallis
Vitamin D (nmol/L)	20.0 (17.0–30.0)	55.0 (18.7–85.5)	—	0.301	Mann–Whitney U
ALP	361 (293–1372) U/L	20.0 (8.8–35.0) U/L	18 (8.3–28.6) units	^ǂ^	
Abnormal skeletal				0.074	Fisher–Halton
Abnormal	7		15		
Normal	1		2		
Not available	3		0		
Bone marrow fibrosis	11/11 (variable degrees)	8/8 (variable degrees)	Biopsy in 5/17 (4/5 significant fibrosis, 1/5 minimal fibrosis)		
Parathyroid histology				0.573	Fisher–Halton
Adenoma	9	8	15		
Hyperplasia	0	0	2		
Carcinoma	1	0	0		
Not available	1	0	0		
Haematological recovery, *n*/*N* ^‡^	9/9 with follow‐up recovered (median 3 [2.5–5] months)	6/6 with repeated biopsy recovered; 2 not repeated	6/7 with follow‐up recovered (3–12 months)		

*Note:* Data are presented as mean ± SD, median (IQR) or number (%), as appropriate. Blank cells indicate data not reported in the original publication.

Abbreviations: ALP = alkaline phosphatase, Hb = haemoglobin, IQR = interquartile range, PTH = parathyroid hormone, SD = standard deviation, Vit D = 25‐hydroxyvitamin D.

^∗^PTH was compared only between case reports and Bhadada et al., as Boxer et al. used assay‐specific units and reference ranges (< 10.0 U/L).

^ǂ^ *p* value for ALP was not calculated due to inconsistencies in reporting units and reference ranges across cohorts.

^‡^Haematological recovery reflects resolution of cytopenias based on available follow‐up data, as reported in the original studies, including peripheral blood parameters and, where available, bone marrow findings.

Haemoglobin concentration showed a highly significant global difference (*p* < 0.001), with the case‐report group demonstrating more severe anaemia (7.6 ± 1.8 g/dL) than the Bhadada (11.5 ± 0.7 g/dL) and Boxer cohorts (9.8 ± 1.2 g/dL). Serum creatinine also differed significantly between groups (*p* = 0.002), with higher values observed in the case‐report group (median: 141.4 μmol/L [IQR: 82–151.5]) and the Boxer cohort (median: 123.8 μmol/L [IQR: 92.8–163.6]) than in the Bhadada cohort (median: 74.9 μmol/L [IQR: 50–74.98]). Hypercalcaemia was a consistent biochemical finding among all groups; however, serum calcium levels differed significantly (*p* < 0.001), with higher mean concentrations in the case‐report group and the Boxer cohort compared to the Bhadada cohort.

Common to the 11 case reports and the two cohorts was a markedly elevated PTH, approximately 10–15 times the upper reference range, with mean concentrations of 130 ± 95.8 pmol/L in the case‐report group, 110.6 ± 102.6 pmol/L in the Bhadada cohort and 99.5 ± 26.4 units in the Boxer cohort. As PTH values in the Boxer cohort were reported in nonstandard assay‐specific units, statistical comparisons were limited to the case‐report group and the Bhadada cohort, which showed no significant difference (*p* = 0.633).

Serum phosphate levels differed significantly between groups (*p* = 0.013), with the lowest mean concentration observed in the case‐report group (0.70 ± 0.10 mmol/L). Vitamin D concentrations were low in the Bhadada cohort (median: 55.0 nmol/L [IQR: 18.7–85.5]) and in the majority of the case‐report group with available data (median: 20.0 nmol/L [IQR: 17.0–30.0]), with no statistically significant difference (*p* = 0.301). Vitamin D values were not reported in the Boxer cohort. ALP was consistently elevated in the case‐report group (median: 361 U/L [IQR: 293–1372]). Although absolute values seemed lower in the Bhadada (median: 20.0 U/L [IQR: 8.8–35.0]) and Boxer cohorts (median: 18.0 units [IQR: 8.3–28.6]), they were nonetheless elevated relative to their respective reference ranges (3–13 U/L for Bhadada and < 4.5 units for Boxer). In the Bhadada cohort, the reported ALP reference range (3–13 U/L) is unusually low and at odds with conventional reference ranges (typically 30–120 U/L). Attempts have been made to contact the authors to exclude a typographical error, but no response was received. Therefore, direct comparisons across cohorts are limited by discrepancies in reporting units and reference values.

Of the 11 case reports, skeletal radiographs were available for 8, of which 7 showed abnormalities. In the Boxer cohort, 15 of 17 cases had abnormal radiographs. Lytic lesions were reported exclusively in the case reports, whereas bone cysts predominated in the Boxer cohort (10/17). Subperiosteal resorption was observed in both groups. Radiographic data were not available for the Bhadada cohort.

Bone marrow biopsies revealed varying degrees of fibrosis in all cases in the case‐report group and the Bhadada cohort, and in all 5 biopsied Boxer cases. Parathyroid adenoma predominated among all groups, having been identified in 9 of 11 cases in the case‐report group, all 8 cases in the Bhadada cohort and 15 of 17 cases in the Boxer cohort, with only one carcinoma in the case‐report group and 2 cases of hyperplasia in the Boxer cohort. Among the 22 cases with available follow‐up data from the case‐report group and the two cohorts, haematological recovery or improvement was observed in 21 cases (95.5%).

Assessment of bone marrow recovery among the various groups differed; in the case‐report group and the Boxer cohort, it was based on normalisation of peripheral blood counts, whereas the Bhadada cohort incorporated repeat bone marrow biopsies. Recovery typically occurred within 2–12 months following treatment.

Where global differences were significant, post hoc pairwise comparisons were presented, as shown in Supporting Table S1.

## 4. Discussion

The predominant clinical characteristics of cases with PHPT‐associated cytopenia are that they are mostly female, present in the fifth decade of life, are almost always symptomatic and have invariably markedly elevated PTH levels. For the most part, cases have inadequate vitamin D levels, and serum ALP is mostly significantly elevated. Commonly reported symptoms are fatigue, weakness and bone pain.

Although the duration of symptoms was reported in only 6 of the 11 cases, it varied widely, from approximately 2 weeks to 36 months. Bone marrow fibrosis and cytopenia were observed across this spectrum, suggesting that prolonged disease may modulate risk but is not a prerequisite for marrow involvement. In contrast, all cases were characterised by markedly elevated PTH levels and biochemical derangements, suggesting that disease severity rather than duration is the dominant determinant of haematological involvement.

The 11 case reports consistently demonstrated bone marrow fibrosis as a key pathological feature in patients with PHPT‐associated cytopenia. Haematological presentations showed considerable variation, ranging from isolated anaemia to more complex patterns such as bicytopenia and pancytopenia, highlighting the heterogeneous nature of bone marrow involvement in PHPT.

Splenomegaly and hepatosplenomegaly were observed in a subset of cases and were thought to reflect extramedullary haematopoiesis. Symptomatic hypercalcaemia was reported in several patients, presenting with polyuria, polydipsia, gastrointestinal disturbances or renal calculi. Haematological abnormalities associated with bone marrow involvement were incidentally identified during routine laboratory testing (Table 1).

Across the 11 cases, skeletal imaging was available in 8, of which 7 were abnormal, demonstrating features of advanced high‐turnover bone disease, including osteopenia, subperiosteal bone resorption, salt‐and‐pepper skull appearance, lytic lesions and brown tumours. Bone marrow fibrosis was a uniform finding, present even in the case with normal radiographs and in cases without imaging, indicating that it can occur independently of overt skeletal disease. While OFC and marrow fibrosis frequently coexisted in severe PHPT, they appear to represent distinct tissue responses to sustained PTH excess. OFC reflects high‐turnover skeletal remodelling driven by prolonged PTH‐mediated osteoclastic activation and fibro‐osseous replacement of trabecular bone, whereas marrow fibrosis involves expansion of fibrotic stroma and reticulin deposition within the marrow niche [24, 25].

Most cases of PHPT are sporadic; approximately 10% have a hereditary basis, including syndromic forms such as MEN1, MEN2A, MEN4 and HPT–jaw tumour (HPT‐JT) syndrome, the latter of which is caused by germline CDC73 mutations [3]. Current guidelines recommend considering genetic testing in patients with familial disease, syndromic features, recurrent or multiglandular PHPT or in sporadic cases diagnosed before the age of 50 [3]. In our case‐report group, 9 of 11 cases were younger than 50 years (ages 15–49), and one patient presented with a mandibular brown tumour, a feature that may raise suspicion for HPT‐JT syndrome. Although genetic data were not available in the 11 case reports, including our own cases, due to financial constraints, genetic analyses for these patients remain a pivotal investigation given their relatively young ages of presentation.

The postulated mechanism for bone marrow fibrosis in PHPT is excessive PTH‐mediated stimulation of fibroblasts. PTH influences marrow stromal and mesenchymal cell populations, altering their proliferation and distribution within the bone marrow [26]. Experimental models have shown that PTH infusion can induce fibroblast proliferation and peritrabecular marrow fibrosis [27].

The degree of serum PTH elevation appears to be a critical determinant of bone marrow involvement. In the 11 cases, PTH levels were markedly elevated, often exceeding 15–20‐fold of the upper reference range, indicating a more aggressive biochemical profile than typically observed in PHPT without marrow involvement. Sustained PTH excess may directly contribute to fibrosis by stimulating stromal fibroblasts, modulating cytokine expression [28] and promoting mesenchymal stem cells’ proliferation and redistribution via PTH receptor signalling [29]. PTH also induces interleukin‐6 (IL‐6) production by osteoblasts [30] and tumour necrosis factor‐α (TNF‐α) expression in extra‐osseous tissues [31]; TNF‐α upregulates Platelet‐derived growth factor‐A (PDGF‐A), a potent fibroblast‐activating cytokine that drives stromal expansion and reticulin deposition [32]. Beyond cytokines, osteocytes mediate PTH signalling through secretion of osteokines such as sclerostin, osteopontin and Fibroblast growth factor 23 (FGF23), which regulate marrow niche homeostasis [33, 34]. Dysregulated osteokine output under chronic PTH excess may perturb stromal behaviour and extracellular matrix turnover, contributing to ineffective haematopoiesis and cytopenia in severe PHPT.

Additional support for the role of PTH‐dependent bone marrow interactions comes from thalassemia models, where impaired PTH signalling in osteolineage cells disrupts the marrow niche, leading to defective haematopoietic stem cell quiescence and reduced self‐renewal. These abnormalities are associated with altered osteocyte‐ and stromal‐derived factors such as osteopontin and Jagged‐1 and are reversible with restoration of PTH signalling [35]. Although pathogenetically distinct from PHPT, these findings underscore the sensitivity of haematopoiesis to disturbances in PTH–bone marrow crosstalk and provide a supportive mechanistic context for marrow involvement in PHPT.

Another nearly invariable finding was marked elevation of serum ALP (median: 361 U/L, IQR: 293–1372 U/L), a surrogate marker of osteoblastic activity reflecting high‐turnover bone remodelling driven by chronic PTH excess [36]. In several cases, elevated ALP levels corresponded to significant skeletal involvement, corroborated by characteristic findings on radiographs and DEXA scans.

Vitamin D deficiency plays a significant role in the severity and clinical presentation of PHPT. Low 25‐hydroxyvitamin D (25[OH]D) levels exacerbate PTH overproduction, resulting in higher circulating PTH levels [37], and are consistently associated with elevated bone turnover markers, lower BMD and high‐turnover bone disease, such as OFC [38, 39]. Observational and cohort data further reinforce this association, showing that symptomatic PHPT is more prevalent in regions with endemic vitamin D deficiency and is more often accompanied by advanced biochemical and skeletal manifestations [38, 39]. In the PHPT‐associated cytopenia cases reviewed, although vitamin D measurements were not uniformly available, the assessed levels were low, suggesting that deficiency may have contributed to both the severity of clinical and biochemical manifestations and haematological involvement, including marrow fibrosis and cytopenia.

Hypophosphataemia, a frequent metabolic consequence of PTH excess, may represent an underrecognised contributor to anaemia in PHPT. Phosphate depletion disrupts erythrocyte energy metabolism by reducing intracellular adenosine triphosphate (ATP) and 2,3‐diphosphoglycerate synthesis, thereby increasing red‐cell rigidity and susceptibility to haemolysis [40]. These metabolic effects may exacerbate anaemia in the setting of severe PHPT, particularly when coexisting with bone marrow involvement.

When comparing the 11 case reports with the Bhadada and Boxer cohorts, bone marrow fibrosis was consistently observed among all patients who underwent biopsy. Despite this shared histopathological feature, the haematological presentations were markedly distinct. The cohorts reported only isolated anaemia, without accompanying leucopenia or thrombocytopenia. In contrast, the case reports demonstrated a broader, more severe spectrum of cytopenias, including pancytopenia, bicytopenia and isolated anaemia with significantly lower haemoglobin concentrations (Table 1). The reason for this variation in presentation is unclear and may reflect the heterogeneous nature of published case reports compared to cohort data.

Although ALP was significantly elevated in all 3 groups, direct comparisons were not feasible due to inconsistent reporting. The Boxer cohort presented values in an unspecified, nonstandard unit with a stated upper reference limit of < 4.5 units [17], whereas the Bhadada cohort reported ALP in U/L but applied an unusually low reference range (3–13 U/L) [16]. Notably, low 25(OH)D concentrations were observed in both the Bhadada cohort and the case‐report group, consistent with a potential link between PHPT, marrow involvement and cytopenia. Vitamin D levels were not reported in the Boxer cohort.

Overall, despite the variations observed among the case‐report group and the two cohorts, consistent findings included a female predominance, elevated serum calcium, PTH and ALP levels, the presence of bone marrow fibrosis and a predominance of parathyroid adenomas. Abnormal radiographic findings were also frequent, though imaging data were limited to the case‐report group and the Boxer cohort.

Bisphosphonates are recommended for patients with PHPT who have low BMD or osteoporosis, especially those who are not candidates for parathyroid surgery, as these agents improve BMD and lower the risk of fragility fractures by inhibiting osteoclast‐mediated bone resorption [3]. Emerging evidence suggests that bisphosphonates also influence the bone marrow microenvironment and haematopoietic stem cells (HSCs), revealing a complex interplay between bone remodelling, osteoblast and osteoclast activity and haematopoiesis. This interaction is particularly relevant in PHPT‐associated cytopenia, underscoring both therapeutic potential and challenges in balancing skeletal health with haematopoietic function. Zoledronic acid has been shown to expand HSC populations by modulating the osteoblastic niche and promoting self‐renewal by upregulating genes, such as Bmi‐1 and Ink4a [41]. However, these effects must be balanced against evidence that bisphosphonate‐induced osteoclast inhibition may impair HSC function, leading to reduced hematopoietic progenitor cell proportions, diminished engraftment capabilities and disruption of normal hematopoietic balance [42].

The prognosis for patients with PHPT‐induced cytopenia is generally favourable, particularly following parathyroidectomy. In our analysis of the 11 cases, haematological recovery was observed in 9 cases, with a median recovery time of 3 months (IQR: 2.5–5.0) following surgery. Recovery data were not available for the remaining 2 cases, precluding assessment of their haematological outcomes. Bhadada et al. and Boxer et al. reported variable recovery times. Differences in disease duration and the extent of marrow fibrosis likely contribute to this variation. Although we have no proof, we postulate that early diagnosis, correction of metabolic derangements of PHPT and timely surgical intervention may improve haematological outcomes. Our evaluation of the data indicates that parathyroidectomy is the only definitive treatment for PHPT‐induced cytopenia.

This study has limitations that impact the strength and generalisability of its findings, largely reflecting the rarity of this clinical entity. The small sample size (11 case reports and 2 small cohorts) and the retrospective nature of the reports restrict external validity. Data completeness was variable across the studies; notably, in the Boxer cohort, bone marrow biopsy findings were available for only 5 of 17 cases, limiting histopathological comparisons. Nuclear imaging data were not available but could have contributed to the understanding of metabolic changes, including increased bone turnover, presence of brown tumours and focal involvement that would otherwise not be detected. The absence of a control group comprising PHPT without cytopenia limits the assessment of risk factors specific to haematological involvement. Inconsistent reporting of key biochemical parameters, particularly serum ALP and PTH, and the absence of vitamin D data in several cases, further limit generalisability.

## 5. Conclusions

This review underscores the association between PHPT and cytopenia due to bone marrow fibrosis, highlighting markedly elevated PTH and ALP levels, low vitamin D levels and skeletal involvement as key determinants of haematological manifestations. While symptom duration may modulate the risk, disease severity appears to be the main determinant driving marrow involvement. In contrast to cohort studies that predominantly reported isolated anaemia, the case‐report group demonstrated a broader spectrum of cytopenias, including bicytopenia and pancytopenia, consistent with more extensive haematological involvement. Importantly, haematological derangements are generally reversible following parathyroidectomy, reinforcing the value of early recognition and timely surgical intervention. Prospective studies with standardised biochemical, skeletal and marrow assessments are warranted to define predictors, risk factors and outcomes of PHPT‐associated cytopenia and marrow fibrosis.

## Funding

No funding was received for this study.

## Conflicts of Interest

The authors declare no conflicts of interest.

## Supporting Information

Additional supporting information can be found online in the Supporting Information section.

## Supporting information


**Supporting Information** Supporting Table S1 presents the post hoc pairwise comparisons for variables showing significant global differences.

## Data Availability

The data that support the findings of this study are available from the corresponding author upon reasonable request.
