# Supplementary material for: Biochemical Predictors and Clinical Characteristics for the Development of Cytopenia and Bone Marrow Involvement Among Patients With Primary Hyperparathyroidism
Source: Int J Endocrinol. 2026 Jul 27;2026:4158433. doi: 10.1155/ije/4158433 (PMC13403217; doi:10.1155/ije/4158433)
Supplement: Supplementary file 1 — Supporting Information Supporting Table S1 presents the post hoc pairwise comparisons for variables showing significant global differences. [file IJE-2026-4158433-s001.docx]

**Supplementary Table S1**. Pairwise Comparisons for Variables with Significant Global Differences

| Variable | Case reports vs  Bhadada | Case reports vs  Boxer | Bhadada vs  Boxer | Test |
| --- | --- | --- | --- | --- |
| Age (years) | 0.778 | 0.004 | <0.001 | Mann–Whitney U |
| Hemoglobin (g/dL) | <0.001 | 0.009 | <0.001 | Games–Howell |
| Serum calcium  (mmol/L) | 0.003 | 0.910 | <0.001 | Games–Howell |
| Serum phosphate  (mmol/L) | 0.011 | 0.129 | 0.435 | Bonferroni |
| Serum creatinine  (µmol/L) | 0.006 | 0.704 | <0.001 | Mann–Whitney U |

Holm‑adjusted significance thresholds for Mann–Whitney U comparisons were as follows:

- Age — Case Reports vs Bhadada 0.050, Case Reports vs Boxer 0.025, Bhadada vs Boxer 0.0167
- Serum creatinine — Case Reports vs Bhadada 0.025, Case Reports vs Boxer 0.050, Bhadada vs Boxer 0.0167

P‑values lower than the corresponding Holm‑adjusted threshold were considered statistically significant.
